# A Multicenter Prospective Protocol to Explore the Relationship Between *Enterococcus faecalis* Bloodstream Infections and Colorectal Neoplasms (EnteroColonus-GAMES Project)

**DOI:** 10.3390/microorganisms14102178

**Published:** 2026-09-28

**Authors:** Juan M. Pericàs, María A. Cañas, Miguel A. Goenaga, Claudia Nevado, Martha Kestler, Sofía De La Villa, Patricia Muñoz, Rosa Blanes, María Tasias, Rosa Escudero-Sánchez, Javier Moreno-García, M. Mar Alonso-Socas, Ángeles Rodríguez-Esteban, Jorge Calderón-Parra, Itziar Diego-Yagüe, Lucía Boix-Palop, Lara García-Álvarez, Oriol Gasch, Oriol Careta, Roger Paredes, Irina S. Luzko, Cristina Garcia-De-La-Mària, Marta Hernández-Meneses, Guillermo Cuervo, Asunción Moreno, Cesar A. Arias, José M. Miró

**Affiliations:** 1 Liver Unit, Vall d’Hebron University Hospital, Vall D’Hebron Institute for Research, Universitat Autònoma de Barcelona, 08035 Barcelona, Spain; 2 Liver and Digestive Diseases (CIBERehd), Spanish Network of Biomedical Research Centers, 08028 Barcelona, Spain; 3 Hospital Clínic of Barcelona-IDIBAPS, University of Barcelona, 08036 Barcelona, Spain; 4 IIS Biogipuzkoa, Department of Infectious Diseases, Research Group on Infectious Diseases and Global Health, Osakidetza, Hospital Universitario Donostia, Infectious Diseases Unit, 20014 San Sebastian, Spain; 5 Department of Clinical Microbiology and Infectious Diseases, Hospital General Universitario Gregorio Marañón, Instituto de Investigación Sanitaria Gregorio Marañón (IiSGM), 28009 Madrid, Spain; 6 CIBERES, Instituto de Salud Carlos III, 28029 Madrid, Spain; 7 Department of Infectious Diseases, Hospital De La Fe, 46026 Valencia, Spain; 8 Department of Infectious Diseases, Ramón Y Cajal University Hospital, Ramón Y Cajal Health Research Institute (IRYCIS), 28034 Madrid, Spain; 9 CIBERINFEC, Instituto de Salud Carlos III, 28029 Madrid, Spain; 10 Hospital Universitario de Canarias, 38320 Tenerife, Spain; 11 Intensive Caresss Unit for Cardiology, Hospital Universitario Central de Asturias, 33011 Oviedo, Spain; 12 Infectious Disease Unit, Hospital Universitario Puerta De Hierro-IDIPHISA Majadahonda, 28222 Madrid, Spain; 13 Department of Infectious DIseases, Hospital Universitari Mútua Terrassa, 08221 Barcelona, Spain; 14 Hospital San Pedro-CIBIR, 26006 Logrono, Spain; 15 Parc Taulí Hospital Universitari, Institut D’Investigació i Innovació Parc Taulí I3PT, Parc Taulí, 08208 Sabadell, Spain; 16 IrsiCaixa, Hospital Germans Trias i Pujol, 08916 Badalona, Barcelona, Spain; ocareta@irsicaixa.es (O.C.);; 17 Department of Medicine, Division of Infectious Diseases, Houston Methodist Hospital, Houston, TX 77030, USA; 18 Reial Academia De Medicina De Catalunya (RAMC), 08001 Barcelona, Spain

**Keywords:** *Enterococcus faecalis*, bacteremia, infective endocarditis, colorectal neoplasia, colonoscopy, microbiome, protocol

## Abstract

Preliminary evidence suggests that patients with *Enterococcus faecalis* bacteremia and infective endocarditis, particularly those without an identifiable source, may have an increased prevalence of colorectal neoplasia. Whether systematic colonoscopy should be recommended in this population remains unresolved. EnteroColonus is a multicenter, prospective, observational matched cohort study that will recruit adults aged 50 years or older with *E. faecalis* bacteremia and/or infective endocarditis from 22 Spanish centers, together with asymptomatic controls undergoing colorectal cancer screening. Cases will be stratified into *E. faecalis* bacteremia/endocarditis of unknown origin (EFBEUO) and with suspected source (EFBESS). The original planned sample size initially was 414 participants: 276 cases and 138 matched controls. Under the amended protocol, the current confirmatory sample-size target is 165 participants (110 cases and 55 matched controls; 2:1 case-to-control allocation). Core procedures include colonoscopy, histopathology, clinical and microbiological phenotyping of colonic samples, fecal microbiome profiling by shotgun metagenomics, detection of *E. faecalis* genetic material in colorectal biopsy specimens using 16S sequencing and species-specific qPCR, and whole-genome, SNP, and phylogenetic analyses of clinical isolates. Secondary endpoints include microbiome profiles, biopsy positivity for *E. faecalis*, genomic traits of bloodstream isolates, mortality, relapse/recurrence, and development of a colorectal neoplasia risk score. This protocol is designed to clarify which patients with *E. faecalis* bloodstream infection might benefit from systematic colonoscopy evaluation and to explore host–microbe mechanisms underlying the observed association with colorectal neoplasia.

## 1. Introduction

Enterococci have emerged over recent decades as leading causes of bloodstream infection in industrialized settings, particularly among older and medically complex patients [1,2,3]. At the same time, colorectal cancer (CRC) remains a major cause of cancer burden and death [4,5]. The best-established infection–neoplasia paradigm in this field is the association between *Streptococcus gallolyticus* bacteremia/endocarditis and colorectal neoplasia, for which colonoscopic evaluation is widely endorsed [6,7,8,9,10,11]. Against this background, accumulating reports have suggested that *Enterococcus faecalis* bloodstream infection may also signal occult colorectal lesions, especially when no clear portal of entry is identified [12,13,14,15,16,17,18].

The EnteroColonus rationale stems from preliminary findings showing a markedly increased prevalence of advanced adenomas and colorectal carcinoma among patients with *E. faecalis* infective endocarditis of unknown origin who underwent colonoscopy [12,13,14]. Additional case reports and small series have described colonic abnormalities in patients with enterococcal infective endocarditis or bacteremia [15,16,17,18], while experimental literature suggests that *E. faecalis* may contribute to colonic carcinogenesis through oxidative stress, extracellular superoxide generation, inflammatory signaling, and interactions with gut dysbiosis [19,20,21]. Moreover, recent evidence from large-scale metagenomic studies has identified specific microbial signatures enriched in colorectal cancer patients, supporting a broader role for the microbiome in colorectal carcinogenesis [22,23,24]. The project summary also notes that diagnosis of CRC can occur during follow-up after the initial infectious episode, raising the possibility that bloodstream infection may function as a clinical warning sign of otherwise unrecognized colorectal neoplasia [13,18].

Despite these signals, major uncertainties remain. It is not known whether the excess risk is confined to infective endocarditis or extends to bacteremia without endocarditis; whether it is limited to episodes without an identifiable source; which types of colorectal lesions are enriched; whether microbiome patterns differ across patient groups; whether colorectal tissue demonstrates enrichment with *E. faecalis* genetic material; and whether particular *E. faecalis* strains harbor genomic features associated with translocation, virulence, antimicrobial resistance, or colorectal neoplasia [13,16,17,18,19,20,21]. There is also no validated clinical model to guide selective colonoscopy after *E. faecalis* bloodstream infection.

This manuscript describes the protocol of the ongoing prospective EnteroColonus study. The objectives of this research project are as follows: The primary objective is to prospectively study patients with EFBEUO by colonoscopy and compare endoscopic/histopathologic findings with those of patients with EFBESS and matched asymptomatic individuals undergoing CRC screening. Secondary objectives are to compare fecal microbiome profiles across groups; assess the presence of *E. faecalis* genetic material in colorectal biopsy samples; compare genomic characteristics of bloodstream isolates according to source classification and colonoscopic findings; and develop a risk score for colorectal neoplasia among patients with *E. faecalis* bloodstream infection.

## 2. Methods

### 2.1. Study Design and Setting

EnteroColonus is designed as a multicenter, prospective, observational matched cohort study. Recruitment will be performed across 22 Spanish centers, with biospecimen processing centralized through Hospital Clínic Barcelona and partner laboratories including IrsiCaixa for microbiome and biopsy-associated molecular analyses and the Center for Antimicrobial Resistance and Microbial Genomics in Houston, Texas, for isolate genomics. The project duration in the original plan was 3 years (Figure 1). Recruitment commenced in 2021 and was ongoing as of September 2026; the recruitment period was extended because of pandemic-related disruptions and slower-than-anticipated case accrual.

Cases comprise adults aged 50 years or older with *E. faecalis* bacteremia and/or infective endocarditis, classified by source as unknown origin (EFBEUO) or suspected source (EFBESS). Controls are asymptomatic individuals aged 50 years or older undergoing screening colonoscopy, matched to EFBEUO cases by site, sex, and age within ±2 years.

### 2.2. Recruitment and Eligibility Criteria

Eligible cases are patients aged 50 years or older with an episode of *E. faecalis* bacteremia and/or infective endocarditis and at least 6 months of follow-up.

Infective endocarditis is defined according to modified Duke criteria [25]. A suspected source is assigned when clinical and/or microbiological findings strongly support a clear portal of entry, such as catheter-related infection, urinary tract source with compatible symptoms and urine culture, surgical wound infection, or biliary focus. Otherwise, the episode is categorized as unknown origin.

Controls are asymptomatic screening participants meeting matching criteria. Exclusion criteria include previous *E. faecalis* bacteremia/endocarditis, previous CRC, familial CRC or hereditary CRC syndromes, and follow-up shorter than 6 months. Table 1 shows the eligibility criteria.

### 2.3. Study Population

The original planned sample includes 414 participants: 276 cases and 138 matched controls. The sample size assumes an annual median of approximately 45 *E. faecalis* infective endocarditis cases in the recruiting network, a bacteremia-to-endocarditis ratio of roughly 3:1 to preserve power for infective endocarditis subgroup analyses, 30–50% of cases with unknown origin, and a relapse rate of 3–8%. Approximately half of the case population is expected to fall within the EFBEUO category. Following the protocol amendment, the current target sample size is 165 participants: 110 cases and 55 matched controls (2:1 case-to-control allocation). This amended target supersedes the original operational estimate. Relapses will contribute clinical data and isolate analyses, whereas recurrences may trigger repeat colonoscopy and repeat fecal sampling if clinically indicated.

### 2.4. Outcome Measures

The primary endpoint is the rate of colorectal neoplasia detected by colonoscopy and confirmed by histopathology in EFBEUO compared with EFBESS and matched controls. Colorectal lesions are first classified endoscopically as neoplastic or non-neoplastic and then histopathologically as malignant (advanced adenomas, colorectal carcinoma, and other colorectal cancers) or non-malignant [13].

Secondary endpoints include fecal microbiome composition and diversity; detection of *E. faecalis* DNA in biopsy material by 16S sequencing and species-specific qPCR; genomic features of bloodstream isolates including WGS profiles, SNPs, phylogenetic clustering, and antimicrobial resistance-related traits; 30-day mortality for bacteremia; 1-year mortality for infective endocarditis; relapse/recurrence; cardiac surgery in infective endocarditis; and clinically relevant treatment changes prompted by colorectal findings. Under the amended protocol, the primary endpoint is confirmatory, whereas all secondary endpoints are exploratory.

### 2.5. Study Procedures and Schedule of Assessments

For cases, baseline data will include epidemiological, clinical, therapeutic, microbiological, and diagnostic information. Colonoscopy will be performed according to routine endoscopic practice, with lesion biopsy when clinically indicated. Fecal samples for microbiome analysis will preferably be collected 48 h before bowel preparation; if not feasible, collection may occur 14 days after colonoscopy using a stabilization system (Table 2).

Table 3 shows the schedule of assessments. All participants will contribute to one primary fecal sample, while repeat samples may be cryopreserved for participants who undergo follow-up colonoscopy. Patients will be followed at approximately 1 month and 6 months after the index admission. Follow-up colonoscopy will only be performed when indicated by routine clinical guidelines (Figure 2).

### 2.6. Data Management

Clinical data will be captured using the established Spanish Collaboration on Endocarditis–Grupo de Apoyo al Manejo de la Endocarditis Infecciosa en España (GAMES) shared database [26] together with a specific case report form for colorectal and biospecimen variables in RedCAP. The source document indicates that the network already maintains standard case report forms (CRFs) and a data manager, and that an additional project/data manager and contract research organization support were planned for coordination, monitoring, and query resolution. Microbiology laboratories at each center will store *E. faecalis* strains locally until shipment. Fecal samples and paraffin-embedded biopsy material will be centralized through Hospital Clínic Barcelona and then distributed to the designated molecular laboratories. This structure supports standardized handling, batched processing, and traceability across centers.

### 2.7. Sample Size Calculation

The original project proposal included an operational recruitment estimate of 414 participants (276 cases and 138 controls), based on the expected number of *Enterococcus faecalis* bacteremia and infective endocarditis episodes in the participating GAMES network, the anticipated proportion of episodes of unknown origin, the expected bacteremia-to-endocarditis distribution, and the possibility of relapse or recurrence. This figure should therefore be interpreted as a feasibility-based recruitment target rather than as the formal minimum sample size required to achieve a prespecified statistical power.

A formal power-based sample size calculation was subsequently undertaken for the primary endpoint, defined as the prevalence of colorectal neoplasia detected by colonoscopy and confirmed by histopathology in patients with *E. faecalis* bacteremia/endocarditis of unknown origin compared with the predefined comparison group. The number of cases was treated as the primary quantity to be estimated, and the number of controls was derived from the prespecified control-to-case allocation ratio.

In the initial confirmatory design, the planned allocation was one control for every three cases. Assuming a two-sided α of 0.05 and 80% power, the resulting formal target sample size was reduced from 414 to 324 participants, corresponding to 243 cases and 81 controls. At that stage, this sample size was considered adequate to address the primary hypothesis while preserving reasonable precision for the main planned secondary clinical comparisons. By contrast, microbiome, tissue-microbiology, and bacterial-genomics analyses were considered exploratory and were not individually powered as confirmatory endpoints.

Recruitment was subsequently affected by the COVID-19 pandemic and, thereafter, by an apparent sustained decrease in the incidence of *E. faecalis* bacteremia/endocarditis in the participating setting, plausibly related to persistent changes in hospital epidemiology and infection-control measures introduced during and after the pandemic. A protocol amendment was therefore introduced to preserve confirmatory testing of the primary hypothesis only, while reclassifying the remaining objectives as secondary or exploratory. Under the amended design, the allocation was revised to one control for every two cases while retaining a two-sided α of 0.05 and 80% power. The final current confirmatory sample-size was 165 participants, corresponding to 110 cases and 55 controls (2:1 case-to-control allocation). This amended target supersedes both the original operational estimate of 414 participants and the initial formal target of 324 participants.

### 2.8. Data Analysis Plan

The analysis plan combines classical epidemiologic methods with multi-omics workflows. The prespecified primary case–control comparison for colorectal neoplasia will constitute the single confirmatory analysis and will be assessed at a two-sided alpha level of 0.05 using a regression model that accounts for the matching factors, with the effect estimate and 95% confidence interval reported. First, descriptive statistics will summarize clinical, endoscopic, histopathologic, microbiological, microbiome, and genomic features. Additional group comparisons will then be conducted using univariate methods appropriate to data type and distribution. For clinical endpoints, logistic regression will be used to identify factors associated with colorectal neoplasia, mortality, and relapse, while Kaplan–Meier curves may be used for time-to-event outcomes. For shotgun metagenomic data, the proposal specifies quality control, decontamination, taxonomic profiling, gene richness estimation, KEGG-based functional profiling, and strain-level characterization, followed by richness/diversity analyses, non-metric multidimensional scaling, correlation testing, pairwise and multi-group nonparametric testing with multiplicity correction, and longitudinal modeling when repeat sampling exists. For isolate genomics, WGS annotation, SNP calling, and phylogenetic analyses will be used to compare strains across exposure groups and lesion phenotypes. Finally, an exploratory multivariable integrated prediction model will be developed to generate a colorectal neoplasia risk score in EFBE, potentially including propensity-score methods and ROC curve assessment. Because the source proposal framed some omics variables as exploratory, this manuscript should be interpreted as a protocol-level analytic plan subject to prespecified statistical refinement before database lock.

### 2.9. Study Strengths and Limitations

To our knowledge, EnteroColonus is the first prospective multicenter study to assess the relationship between *E. faecalis* bacteremia or infective endocarditis and colorectal neoplasia while integrating colonoscopic and histopathologic findings with fecal-microbiome profiling, and whole-genome analysis bloodstream-isolate. The inclusion of source-defined case groups and matched asymptomatic screening controls permits comparisons across clinically relevant populations within a common protocol.

The principal limitation is the difficulty of recruitment. Patients with *E. faecalis* infective endocarditis are often older, and some are unable or unwilling to undergo colonoscopy, which can reduce participation and introduce selection bias. These recruitment constraints, together with pandemic-related disruption, led to recalculation of the sample size. Although the amended sample is intended to test the primary endpoint, it may be insufficient to detect differences in the molecular and microbiome substudies, particularly in analyses stratified by bacteremia versus endocarditis, infection source, or healthcare acquisition. These analyses will therefore be treated as exploratory, and nonsignificant trends will be interpreted cautiously. The observational design also precludes causal inference, and antibiotic exposure, bowel preparation, sampling time, and missing biospecimens may affect microbiome comparisons.

### 2.10. Potential Modifications to the Protocol as a Contingency

The original proposal explicitly recognizes that several variables are exploratory because no prior comprehensive dataset exists in this field. Contingency modifications may therefore include refinement of dependent or intermediate omics variables after pilot analyses; adaptation of biospecimen logistics if pre-colonoscopy stool sampling is not feasible; repeat fecal sampling and repeat colonoscopy only in clinically indicated recurrences; and staged methodological adjustments identified during the pilot phase of sequencing and molecular analyses.

In the amended design, the allocation ratio was changed to one control for every two cases, while maintaining a two-sided α of 0.05 and 80% power. Under this revised framework, the recalculated total sample size was 165 participants, corresponding to 110 cases and 55 controls. From that point onward, only the primary endpoint was considered confirmatory. Secondary analyses, including subgroup analyses by bacteremia versus infective endocarditis, microbiome analyses, detection of *E. faecalis* genetic material in biopsy specimens, isolate genomic analyses, and risk-score development, were regarded as exploratory and hypothesis-generating.

A detailed mathematical derivation of the sample size calculation is provided in the Supplementary Material.

### 2.11. Ethics and Dissemination

According to the source protocol, the Ethics and Research Committee of Hospital Clínic de Barcelona was designated to perform the initial protocol and informed-consent review, after which all participating centers were to obtain local approval before study initiation (HCB/2021/0073). Written informed consent is required for enrollment and biospecimen collection. The study is observational, but colonoscopy, biopsy handling, microbiologic isolate storage, and genomic/microbiome analyses require careful governance of privacy, sample traceability, and data security.

Dissemination plans in the project summary include final reporting, conference presentations, and manuscript drafting, with the coordinating investigators leading the first publication and later substudies assigned across sites. Study findings will be disseminated through peer-reviewed publications and presentations at national and international scientific meetings. The main results manuscript will report the primary study objectives, including the association between *Enterococcus faecalis* bloodstream infection and colorectal neoplasia and the comparative diagnostic yield of colonoscopy across the predefined study groups. Secondary manuscripts will address prespecified ancillary analyses, including microbiome profiles, detection of *E. faecalis* genetic material in colorectal biopsy specimens, genomic characterization of bloodstream isolates, and development of a risk model for colorectal neoplasia. Results will be reported regardless of the direction, magnitude, or statistical significance of the findings.

The coordinating investigators will lead the primary dissemination outputs, and additional publications arising from substudies will be developed with participation from investigators across collaborating centers according to their contributions. Findings will also be shared with participating centers to support interpretation of the results and to inform future research and clinical decision-making regarding colonoscopic evaluation in patients with *E. faecalis* bacteremia or endocarditis.

### 2.12. Expected Results, Relevance and Potential Implications for Clinical Practice and Future Research

The study is expected to validate whether EFBEUO is associated with a substantially higher prevalence of colorectal neoplasia than EFBESS and matched screening controls [12,13,14,15,16,17,18]. It is also expected to define whether this association is confined to unknown-source episodes or extends more broadly across *E. faecalis* bloodstream infections [13,16,17,18]. Recruitment commenced in 2021 and remained ongoing as of September 2026. At that date, 141 participants had been enrolled (93 cases and 48 controls). Recruitment was extended after pandemic-related disruption and slower-than-anticipated case accrual. Control recruitment is expected to be completed by 31 December 2026 and case recruitment by 30 March 2027. Figure 3 summarizes the current recruitment status. Current recruitment is depicted in Figure 3.

At the biological level, EnteroColonus is expected to identify group-specific microbiome signatures, characterize the burden of *E. faecalis* genetic material within colorectal lesions, and determine whether particular bloodstream isolates cluster by genomic patterns associated with neoplasia [19,20,21]. Fecal samples will be collected from patients with *E. faecalis* bloodstream infections of unknown or suspected origin, as well as matched asymptomatic colorectal cancer screening controls, and analyzed using whole-metagenome shotgun sequencing. Taxonomic, functional, and strain-level profiling will be performed through established bioinformatic pipelines including MetaPhlAn4, HUMAnN3, KEGG-based functional annotation, FishTaco, and strain-tracking approaches. In parallel, colorectal biopsy samples obtained during colonoscopy will undergo 16S rRNA amplicon sequencing and *E. faecalis*-specific RT-PCR/qPCR analyses to evaluate bacterial enrichment in neoplastic lesions. Whole-genome sequencing and phylogenetic characterization of bloodstream *E. faecalis* isolates will also be performed, including assessment of genomic traits and antimicrobial resistance patterns. Previous studies have shown that gut microbiome dysbiosis is associated with colorectal carcinogenesis and that distinct microbial signatures may be identified in precancerous lesions [13,27]. Integrated statistical analyses combining microbiome, genomic, histopathological, and clinical variables are expected to identify microbial signatures associated with colorectal lesions and to clarify potential relationships between gut dysbiosis, *E. faecalis* translocation, colorectal carcinogenesis, and clinical outcomes.

If successful, the integrated dataset should support construction of a clinically useful risk score to prioritize colonoscopy work-up in patients with *E. faecalis* bacteremia or infective endocarditis. If the study confirms a high burden of colorectal neoplasia in patients with EFBEUO, it could support systematic or risk-stratified colonoscopy as part of the diagnostic work-up for *E. faecalis* bloodstream infection, analogous in concept to current practice for *S. gallolyticus* [8,9,10,13].

Equally important, a negative or source-restricted result would help avoid unnecessary colonoscopy in lower-risk subgroups [13]. Beyond immediate clinical decision-making, the project provides a translational framework linking bedside phenotypes with microbiome and bacterial genomic data [19,20,21]. That framework may clarify mechanisms of translocation and carcinogenesis, enable externally validated prediction tools, and generate hypotheses for future studies on virulence factors, mucosal colonization, longitudinal cancer risk, and intervention strategies [13,19,20,21].

## Figures and Tables

**Figure 1:**
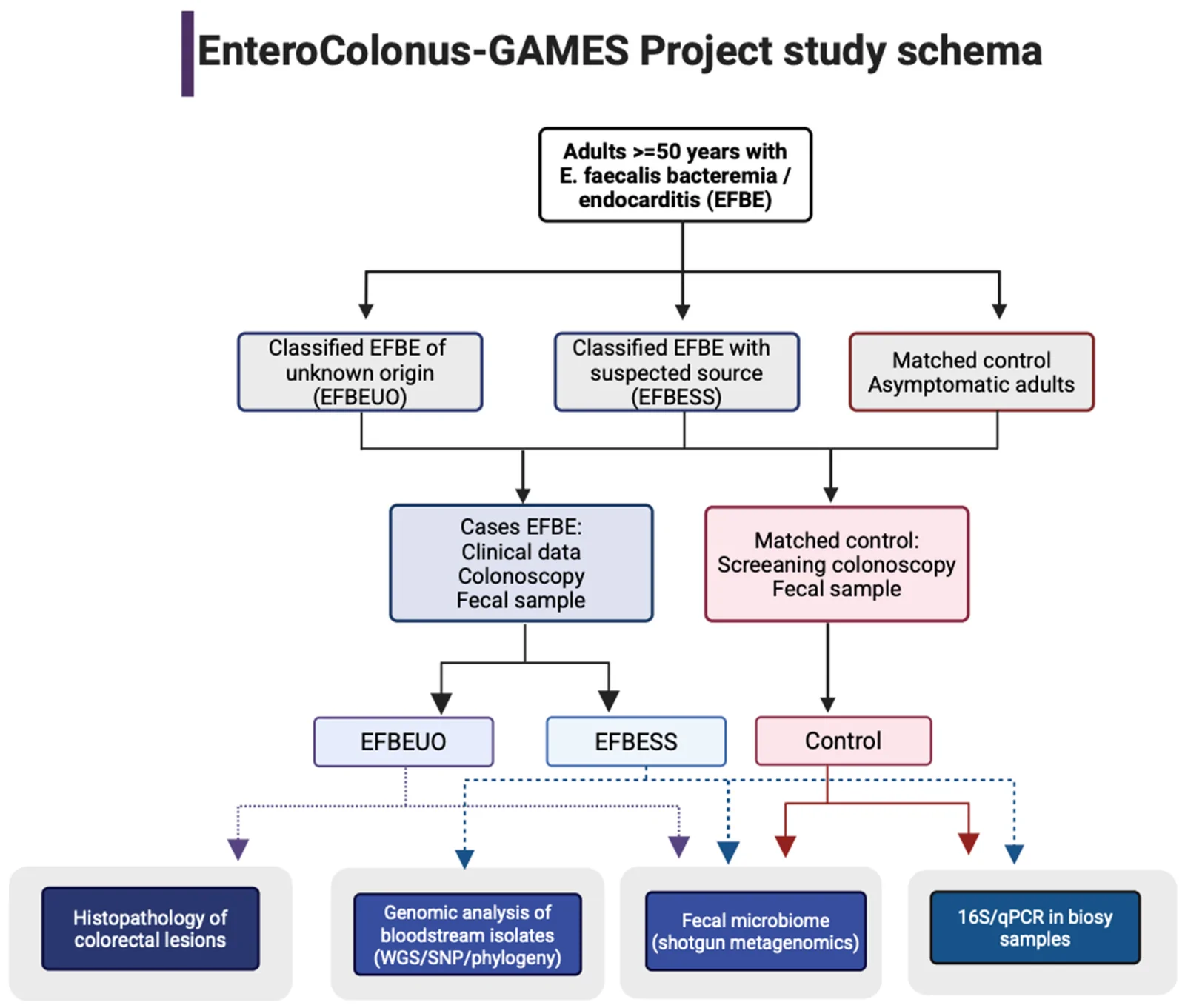
Study design and analytic framework of the EnteroColonus project, showing the three study groups *E. faecalis* bacteremia/endocarditis of unknown origin (EFBEUO) and with suspected source (EFBESS), and matched screening controls; the core clinical and biospecimen procedures; and the downstream histopathologic, microbiome, tissue-microbiology, and bloodstream isolate genomic analyses (qPCR, quantitative polymerase chain reaction; SNP, single-nucleotide polymorphism; and WGS, whole-genome sequencing).

**Figure 2:**
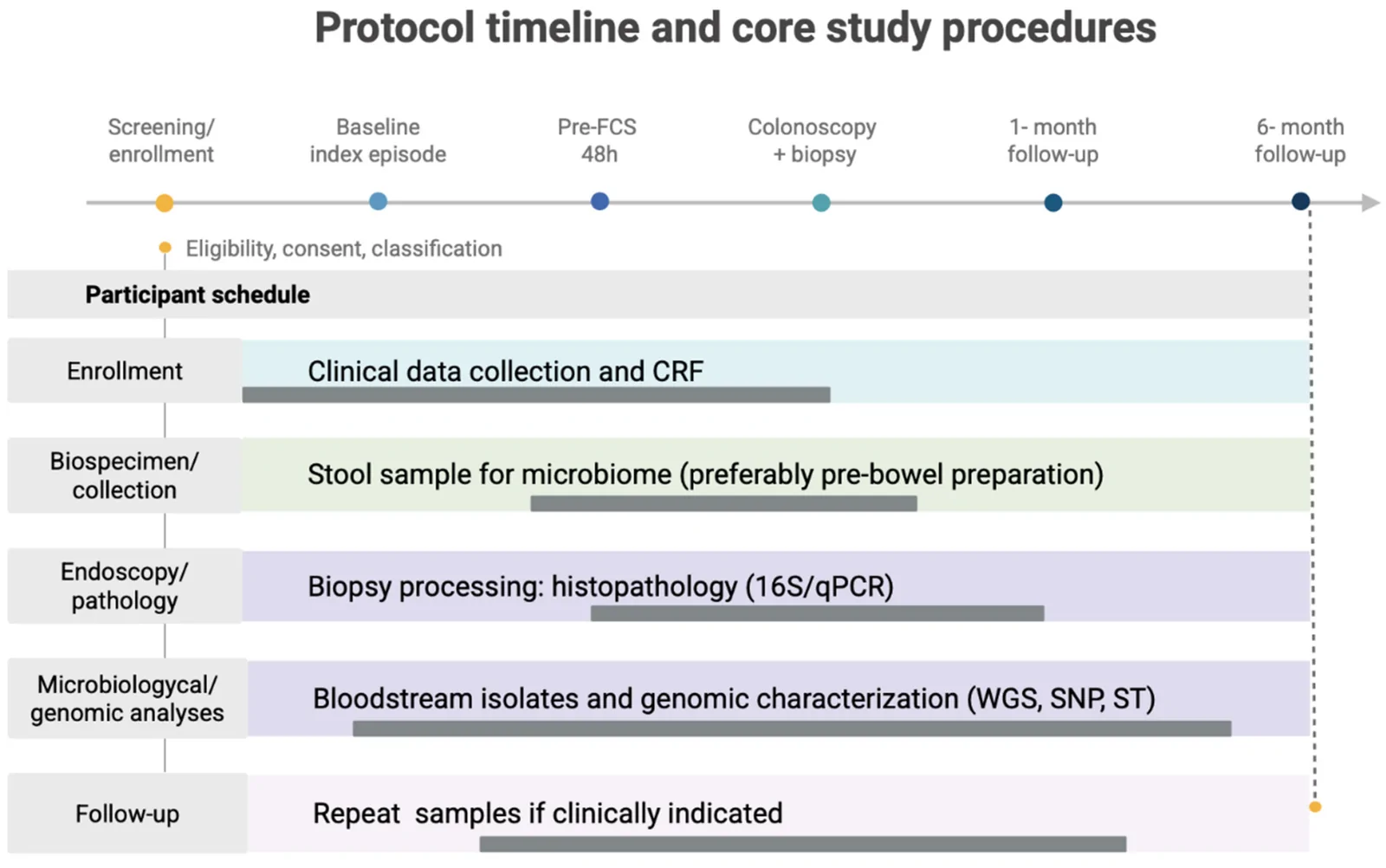
Protocol timeline summarizing the timing of eligibility assessment, biospecimen collection, colonoscopy, and follow-up visits.

**Figure 3:**
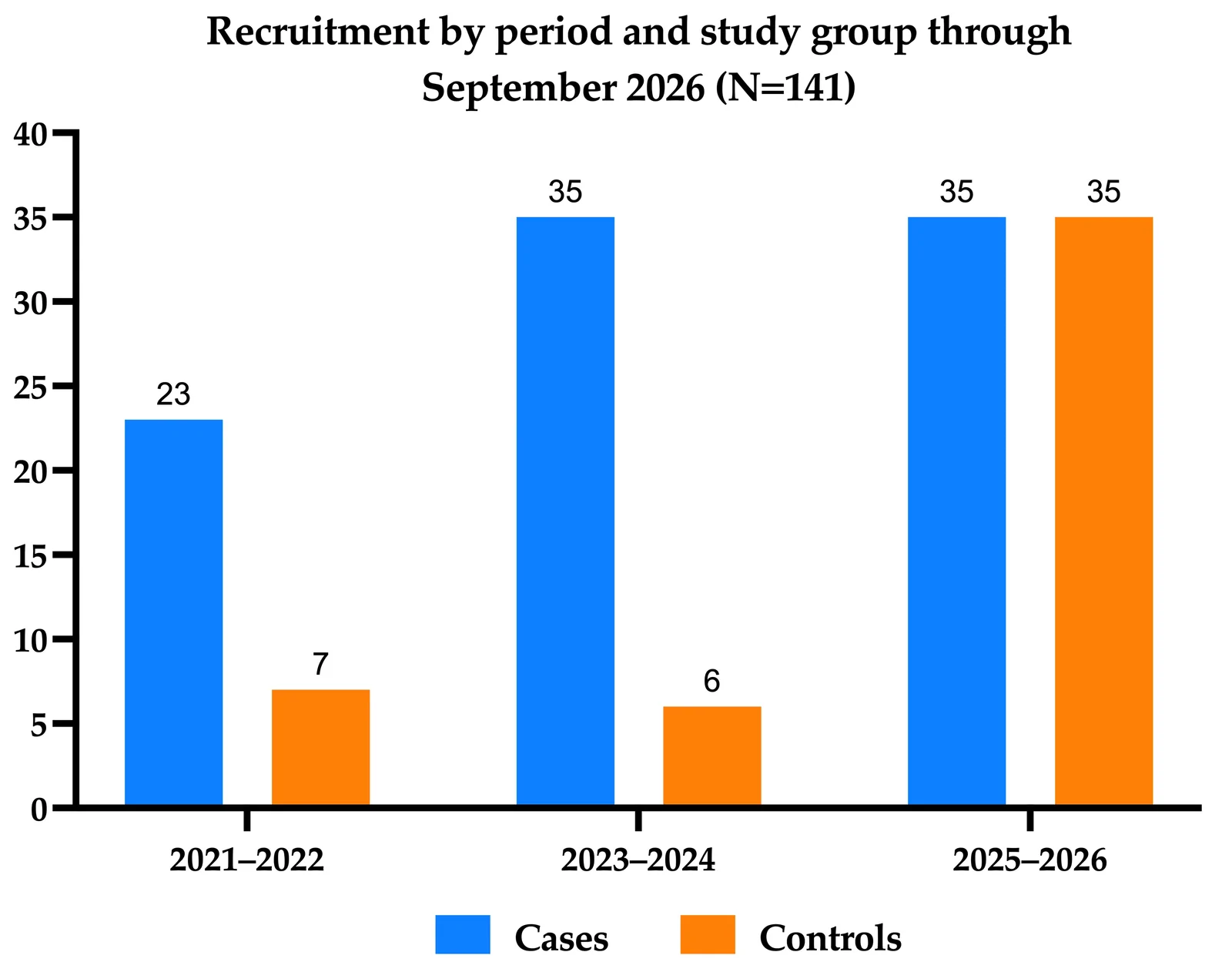
Recruitment of cases and controls as of September 2026. The figure includes 141 participants (93 cases and 48 controls). Control recruitment is expected to be completed by 31 December 2026 and case recruitment by 30 March 2027. Enrollment and all outcome analyses remain ongoing; final participant counts will be reported in the main results article.

**Table 1:** Eligibility criteria and study groups.

	EFBEUO	EFBESS	Matched Controls
**Core definition**	Adults with *E. faecalis* bacteremia and/or infective endocarditis without an identifiable source	Adults with *E. faecalis* bacteremia and/or infective endocarditis with a clinically supported suspected source	Asymptomatic adults undergoing screening colonoscopy for CRC
**Age/follow-up**	Age ≥50 years; ≥6-month follow-up	Age ≥50 years; ≥6-month follow-up	Age ≥50 years
**Matching**	Reference group for matched-control selection	Not matched	Matched to EFBEUO participants by site, sex, and age (±2 years)
**Key exclusions**	Prior EFBE, prior CRC or familial/hereditary CRC syndromes	Prior EFBE, prior CRC or familial/hereditary CRC syndromes	Prior CRC or hereditary/familial CRC syndromes as applicable to local screening practice
**Main study procedures**	Clinical data collection, colonoscopy, histopathology, stool sampling, and isolate genomics	Clinical data collection, colonoscopy, histopathology, stool sampling, and isolate genomics	Screening colonoscopy and stool sampling

CRC, colorectal cancer; EFBE, *E. faecalis* bacteremia/endocarditis; EFBESS, *E. faecalis* bacteremia/endocarditis with suspected source; EFBEUO, *E. faecalis* bacteremia/endocarditis of unknown origin.

**Table 2:** Planned endpoints and analytic domains.

	Endpoint/Measure	Method/Source
**Primary clinical endpoint**	Colorectal neoplasia detected across EFBEUO, EFBESS, and controls.	Colonoscopy with histopathology.
**Microbiome**	Taxonomic composition, richness/diversity, functional pathways, strain-level features.	Shotgun metagenomic sequencing of stool samples
**Tissue microbiology**	Presence and relative enrichment of *E. faecalis* in colorectal lesions.	16S sequencing and species-specific qPCR on FFPE biopsy samples.
**Bacterial genomics**	Genomic annotation, SNPs, phylogeny, resistance/virulence-associated features.	WGS, variant calling and phylogenetic analysis of bloodstream isolates
**Clinical outcomes**	30-day mortality, 1-year mortality, relapse/recurrence, cardiac surgery.	Prospective follow-up and CRF-based data review.
**Prediction model**	Risk score for colorectal neoplasia among EFBE patients.	Integrated clinical, endoscopic, microbiologic, and omics analyses.

CRC, colorectal cancer; CRF, case report form; EFBE, *E. faecalis* bacteremia/endocarditis; EFBESS, *E. faecalis* bacteremia/endocarditis with suspected source; EFBEUO, *E. faecalis* bacteremia/endocarditis of unknown origin; FFPE, formalin-fixed, paraffin-embedded; SNPs, single-nucleotide polymorphisms; WGS, whole-genome sequencing.

**Table 3:** Schedule of assessments.

Assessment	Enrollment/Index Episode	Pre-Colonoscopy	Colonoscopy	1 Month	6 Months
**Eligibility, consent, source classification**	X				
**Clinical, microbiological and treatment data collection**	X			X	X
**Stool sample for microbiome**		X			X *
**Screening/diagnostic colonoscopy**			X		
**Histopathology of lesions**			X **		
**Biopsy 16S sequencing/** ***E. faecalis*** ** qPCR**			X **		
**Bloodstream isolate storage and genomic analyses**	X				
**Relapse/recurrence adjudication**				X	X

* Repeat stool sampling only if clinically indicated. ** Performed when biopsy material is obtained and lesions are available for analysis.

## Data Availability

The original contributions presented in this study are included in the article/Supplementary Material. Further inquiries can be directed to the corresponding authors.

## Supplementary Materials

The following supporting information can be downloaded at https://www.mdpi.com/article/10.3390/microorganisms14102178/s1: Mathematical derivation of the sample size calculation.

## Appendix A. EnteroColonus Investigators

**Hospital Universitario La Princesa (Madrid)**: Carmen Sáez, Javier B Pérez-Serrano, M Auxiliadora Semiglia-Chou, Nelly D Zurita, J Andrés Moreno-Monteagudo, Jorge Mendoza Jiménez-Ridruejo; **Hospital Central de Asturias (Oviedo)**: Mª Ángeles Rodríguez, Raquel Rodríguez, Fernando Fernández-Cadenas, Mª Eugenia Llaneza; **Hospital Mutua Terrassa (Barcelona)**: Lucía Boix, Pablo Ruíz, Beatriz Dietl, Cristina Badía; **Hospital Universitario Puerta de Hierro (Madrid)**: Jorge Calderón-Parra, Antonio Ramos, Itziar Diego-Yagüe, M Isabel Veral-Mendoza; **Hospital Universitario Ramón y Cajal (Madrid)**: Rosa Escudero, Enrique Navas, Pilar Vizcarra, Sofia Parejo, Javier Moreno-García; **Hospital General Universitario Gregorio Marañón (Madrid)**: Sofía de la Villa, Ana Álvarez-Uría, Marina Machado, Martha Kestler, Patricia Muñoz, Julia Serrano-Lobo, Teresa Vicente-Rangel, Iván Adán-Rodríguez, Almudena Burillo, Beatriz Merino-Rodríguez, Sara Rodríguez; **Hospital Universitario de Canarias (Tenerife)**: Mª Mar Alonso-Socas; **Hospital Universitario de Son Espases (Palma de Mallorca)**: Silvia Peláez-Fernández, M Carmen Garrido, M Àngels Ribas, Enrique Ruiz-de-Gopegui, Isabel Amengual, Silvia Peláez, Laura Vidal; **Hospital San Pedro (Logroño)**: Lara García-Álvarez, Concepción García-García, Estíbaliz Corral; **Hospital Universitario Materno-Infantil de Gran Canaria (Las Palmas de Gran Canaria)**: Elena Pisos-Álamo, Violeta Malo-de-Molina, Isabel de Miguel-Martínez, Cristina Carranza; **Hospital Clínic (Barcelona)**: Asunción Moreno, Guillermo Cuervo, Marta Hernández-Meneses, Juan M Pericàs, Jose M. Miró, Leticia Moreira, Coral Sala, Irina Luzco, Mateu Espasa-Soley, Marta Maristany; **Hospital de Donosti (San Sebastián)**: Alazne Aguirre-García, Xabier Intxausti-Picabea, Miguel Á Goenaga, Claudia Nevado; **Hospital Parc Tauli (Barcelona)**: Victor Monsalvez, Andrea Soult, Oriol Gasch, Eva M Pascual-Gonzalez, Eva Martinez-Bauer, Marina Alguacil; **Hospital Universitario y Politécnico La Fe (Valencia)**: Rosa Blanes, Mariona Tasias, María García Campos, Marta Montero, Juan Frasquet; **IrSICaixa, Hospital Germans Trias i Pujol (Badalona, Barcelona)**: Oriol Careta, Francesc Català-Moll, Mariona Parera, Roger Paredes. **Houston Methodist Hospital (Texas, USA)**: Diana Panesso, Cesar A. Arias.
