# Supplementary material for: A Multicenter Prospective Protocol to Explore the Relationship Between Enterococcus faecalis Bloodstream Infections and Colorectal Neoplasms (EnteroColonus-GAMES Project)
Source: Microorganisms. 2026 Sep 28;14(10):2178. doi: 10.3390/microorganisms14102178 (PMC13707825; doi:10.3390/microorganisms14102178)
Supplement: Supplementary file 1 [file microorganisms-14-02178-s001.zip › microorganisms-4464358-supplementary.pdf]

microorganisms

MDPI

Supplementary Material

Mathematical derivation of the sample size calculation

The formal sample size calculation was based on the comparison of two independent proportions for the primary endpoint, assuming a two-sided significance level of 0.05 and a statistical power of 80% ($1-\beta = 0.80$). The number of cases was treated as the primary quantity to be estimated, whereas the number of controls was derived from the prespecified control-to-case ratio.

Let m denote the number of controls per case:

$m = n\_controls / n\_cases$

Accordingly,

$n\_controls = m \cdot n\_cases$

and the total sample size is:

$N = n\_cases + n\_controls = n\_cases\ (1 + m)$

which can be rearranged as:

$n\_cases = N / (1 + m)$

and

$n\_controls = mN / (1 + m)$

Let p1 be the expected prevalence of colorectal neoplasia among cases and p0 the expected prevalence among controls. Under unequal allocation, the pooled prevalence is:

$\bar{p} = (p1 + m\ p0) / (1 + m)$

The required number of cases is then given by:

$n\_cases = [\ z\_(1-\alpha/2)\ \sqrt{((1+m)\ \bar{p}(1-\bar{p}))} + z\_(1-\beta)\ \sqrt{(p1(1-p1) + m\ p0(1-p0))}\ ]^2 / [\ m\ (p1-p0)^2\ ]$

and the required number of controls is obtained as:

$n\_controls = m \cdot n\_cases$

In the initial confirmatory design, the control-to-case ratio was 1:3 (m = 1/3). With a total sample size of 324 participants:

$n\_cases = 324 / (1 + 1/3) = 243$

$n\_controls = (1/3) \times 243 = 81$

In the amended design, the control-to-case ratio was 1:2 (m = 1/2). With a total sample size of 165 participants:

$n\_cases = 165 / (1 + 1/2) = 110$

$n\_controls = (1/2) \times 110 = 55$

Because statistical power in an a priori sample size calculation is a fixed design parameter rather than an observed estimate, confidence intervals are not usually reported for the target power itself. Instead, expected 95% confidence intervals for the assumed group-specific event proportions may be expressed as:

$95\%\ CI(p) = p \pm 1.96\ \sqrt{(\ p(1-p) / n\ )}$

where p is the expected proportion and n the corresponding group sample size.
